# Sparse Sampling of Silence Type I Errors With an Emphasis on Primary Auditory Cortex

**DOI:** 10.3389/fnins.2019.00516

**Published:** 2019-05-31

**Authors:** Francis A. M. Manno, Juan Fernandez-Ruiz, Sinai H. C. Manno, Shuk Han Cheng, Condon Lau, Fernando A. Barrios

**Affiliations:** ^1^Instituto de Neurobiología, Universidad Nacional Autónoma de México, Querétaro, Mexico; ^2^Department of Physics, City University of Hong Kong, Kowloon, China; ^3^Department of Biomedical Sciences, City University of Hong Kong, Kowloon, China; ^4^Facultad de Medicina, Universidad Nacional Autónoma de México, Ciudad de México, Mexico

**Keywords:** sparse sampling fMRI, type I error rates, false positives, auditory cortex, null hypothesis

## Abstract

Sparse sampling functional MRI (ssfMRI) enables stronger primary auditory cortex blood oxygen level-dependent (BOLD) signal by acquiring volumes interspersed with silence, reducing the physiological artifacts associated with scanner noise. Recent calculations of type I error rates associated with resting-state fMRI suggest that the techniques used to model the hemodynamic response function (HRF) might be resulting in higher false positives than is generally acceptable. In the present study, we analyze ssfMRI to determine type I error rates associated with whole brain and primary auditory cortex voxel-wise activation patterns. Study participants (*n* = 15, age 27.62 ± 3.21 years, range: 22–33 years; 6 females) underwent ssfMRI. An optimized paradigm was used to determine the HRF to auditory stimuli, which was then substituted for silent stimuli to ascertain false positives. We report that common techniques used for analyzing ssfMRI result in high type I error rates. The whole brain and primary auditory cortex voxel-wise analysis resulted in similar error distributions. The number of type I errors for *P* < 0.05, *P* < 0.01, and *P* < 0.001 for the whole brain was 7.88 ± 9.29, 2.37 ± 3.54, and 0.53 ± 0.96% and for the auditory cortex was 9.02 ± 1.79, 2.95 ± 0.91, and 0.58 ± 0.21%, respectively. When conducting a ssfMRI analysis, conservative α level should be employed (α < 0.001) to bolster the results in the face of false positive results.

## Introduction

Sparse sampling functional MRI (ssfMRI) refers to the acquisition of imaging volumes interspersed with periods of no data acquisition (silent periods), in contrast to the typical continuous acquisition (Edmister et al., 1999; Hall et al., 1999; Talavage et al., 1999). Sparse sampling experiments are implemented in auditory-related paradigms to avoid acquisition noise during stimulus presentation (Schönwiesner et al., 2007; Norman-Haignere et al., 2013). The optimized data acquisition occurs when BOLD signal change is at its maximum due to the delay of the hemodynamic response (∼4–6 s; Perrachione and Ghosh, 2013). Given the recent evidence of false-positive rates in resting state fMRI data (rsfMRI; Eklund et al., 2016), we examined ssfMRI to determine the prevalence of type I errors under an optimized auditory paradigm.

The sparse sampling paradigm is dependent on different repetition times (TR; Edmister et al., 1999; Hall et al., 1999; Talavage et al., 1999). The first protocols were developed by Hall et al. (1999) which acquired a volume every *TR* = 14 s and Edmister et al. (1999) which acquired a volume every *TR* = 8 s. Subsequently, Bunzeck et al. (2005) and Schwarzbauer et al. (2006) acquired a series of 5 volumes per TR, and Zaehle et al. (2007), Schmidt et al. (2008), and Liem et al. (2012) acquired a series of 3 volumes per TR, termed clustered sampling (Gaab et al., 2003, 2007a,b, 2008). The study by Eklund et al. (2016) aggregated rsfMRI data from three sites: Beijing (*TR* = 2 s, 198 subjects, 225 time-points), Cambridge (*TR* = 3 s, 198 subjects, 119 time-points), and Oulu (*TR* = 1.8 s, 103 subjects, 245 time-points), consisting of different TR and volume numbers per subject. Their study explored the familywise error rates for cluster-wise and voxel-wise inferences, with the null hypothesis of no modulation in blood oxygen level-dependent (BOLD) signal and a mean of zero activation. The authors found conservative voxel-wise, but invalid cluster-wise inference associated with the common parametric methods for functional MRI (fMRI). The current understanding of type I error rates in rsfMRI research warrants the investigation of auditory paradigms for false positives.

Here we asked would a sparse sampling paradigm, with a long TR duration normal for fMRI auditory research, result in significant BOLD signal during the presentation of silent stimuli? In ssfMRI, the model is designed to capture auditory stimuli. In the paradigm of the present study, silent stimuli were presented after an auditory paradigm was optimized; therefore, the experiment was done in two steps. We first optimized the auditory experiment for BOLD activation, and second, we substituted our auditory stimuli for the silent stimuli. We explored the voxel-wise error rates associated with the silent stimuli for whole brain activation and for our region of interest (ROI), the primary auditory cortex. The hemodynamic response function (HRF) model between the experiments was identical and the null hypothesis of no BOLD response, was used for the silent experiment. False positives (type I errors) were finding BOLD response in our silent stimuli assessment. Variables in the generic sparse sampling protocol were manipulated to optimize the paradigm (Perrachione and Ghosh, 2013). We excepted the errors within the primary auditory cortex would mirror the distribution of errors found in the whole brain analysis if the model was unbiased for ROI. We anticipated finding a similar number of errors as found in rsfMRI (Eklund et al., 2016), because the only difference in ssfMRI is the long TR value. Contrary to our assumptions, the results of the present study indicate a high prevalence of type I error at *P* < 0.05 in the voxel-wise analysis. The present study recommends using conservative statistical inference for fMRI in order not to breach the assumptions of the underlying tests. Additionally, as previously recommend (Friston, 2012, 2013; Ingre, 2013; Lindquist et al., 2013), future studies should explore false discovery rates (FDRs) and effect size statistics in ssfMRI paradigms.

## Materials and Methods

**FIGURE 1:**
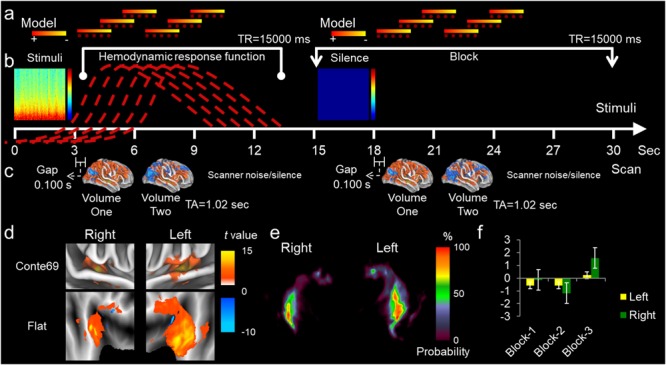
Experimental design. **(a)** General linear model using double gamma function convolved to a canonical hemodynamic response function **(b)**. Where there is a sound stimulus presented, the model evaluates the relationship between our categories (i.e., EVs/regressors) and the dependent variable (BOLD signal). The stimuli are represented as a spectrogram with time on the x-axis and frequency on the y-axis. The color bar represents high frequency in red and low frequency in blue. Each red dashed line represents a putative hemodynamic response function. A model above the red dashed line is represented by a colorbar with high BOLD signal in red (+) and low BOLD signal in yellow (-). For a block of sound presentation, varying the model would tentatively capture the hemodynamic response function. For the presentation of a silent stimulus, seen to the right of the sound stimulus, varying the model should capture resting state or error associated with the model since no sound stimulus exists. **(c)** Below the time line, two volumes were acquired per block separated from the stimuli presentation by gap times from 0.1 to 2.0 s. The TA is 1.02 s and the *TR* = 15,000 ms. Each volume was acquired in 1.02 s and separated from the preceding volume by 1 s. The remaining time within a block was the silent period consisting of background scanner noise in the MRI room (i.e., the helium pump of the cryomagnetic; Hoiting, 2005). **(d)** From the two volume acquisitions, regions of interest were extracted pertaining to Heschl’s gyrus for the left and right hemisphere. The color bar represents activation from 0 to 15 *t*-value and deactivation from 0 to -10 *t*-value. The Heschl’s gyri were extracted based on probabilistic maps **(e)**, where the color bar represents the probability of finding Heschl’s gyrus within that location from 0 to 100%. From the activation/deactivation maps, average *t*-values were calculated **(f)**.

The first series of experiments consisted of optimizing an ssfMRI paradigm based on generic auditory stimuli (Figure 1). The paradigm for auditory stimuli is under review in a subsequent manuscript. Once the paradigm was optimized, the auditory stimuli were substituted for silent stimuli. The experimental paradigms were identical except for the stimuli.

### Study Participants

The study consisted of 15 self-reporting right-handed volunteers age 27.62 ± 3.21 years (range: 22–33 years; 6 females). All volunteers gave informed consent (oral and written) and were free of contraindications for MRI scanning. All individuals were self-reporting right handers, filling forms with the right-hand. Subjects were native Spanish speakers, reporting normal hearing which was confirmed during an initial verbal screening and audio level setting within the scanner. All participants underwent audiometric testing, consisting of presenting and confirming the hearing of a series of pure tones from 400 to 8,000 Hz, in addition to linear sweeps, log sweeps, and white noise in the same frequency range. No subject reported a history of neurological or psychiatric illness. The research protocol was approved by the Comite de Bioética del Instituto de Neurobiología (UNAM) on the Use of Humans as Experimental Subjects in accordance with the Declaration of Helsinki, 2013.

### Stimuli for Optimizing the Sparse Sampling Experimental Design

A variety of “test stimuli” were used in order to assess the HRF and potential activation of auditory cortex. We used three specific stimuli generated with Matlab to activate the auditory cortex during our sparse sampling preliminary study. (1) Linear sweep with frequency range of 440–7,040 Hz at the 16th Harmonic of A4. (2) Log sweep with frequency range of 440–7,040 Hz at the 16th Harmonic of A4. (3) White noise. All stimuli were generated with Matab and tested on a HP pc (Intel Core i5-4210U CPU @ 2.40GHz) with a RealTek High Definition Audio card (Driver Version: 6.0.1.7535) using Stereo Mix (RealTek) Driver. After the “test stimuli” were used to determine an optimal paradigm for the sparse sampling experiment, the silent stimuli were substituted for the previous “test stimuli” and scanning was repeated.

### Image Acquisition

Images were acquired bottom-up interleaved on a 3T MR750 scanner (General Electric, Waukesha, WI, United States). A fast-spoiled gradient echo brain volume imaging (FSPGR BRAVO) was obtained for co-registration, resolution = 1 × 1 × 1 mm^3^, field of view (FOV) = 256 × 256 mm^2^, slice thickness = 1 mm, *TR* = 8.156 s, echo time (*TE*) = 3.18 ms, inversion time (*TI*) = 450 ms, and flip angle = 12°. A single shot gradient-echo echo-planar image (GE-EPI) was used for the fMRI BOLD acquisition with the following parameters: *TR* = 15,000 ms, *TE* = 30 ms, *TA* = 1.02 s, slices = 34, flip angle = 90°, FOV = 256 × 256 mm^2^, matrix = 128 × 128 (yielding voxel size = 2 mm × 2 mm × 3 mm).

### Sparse Sampling Trials to Capture the HRF

To determine the most robust HRF to our 3 s stimuli, a two-volume clustered sparse sampling paradigm was employed, and three variables were optimized for auditory stimuli (Figure 1; Perrachione and Ghosh, 2013). (1) The gap delay, occurring between the end of our 3 s silent stimuli and the beginning of the acquisition time (TA). (2) The duration of the silent period, occurring after the TA and prior to the new stimuli presentation. (3) The TR was the entire block containing the stimulus presentation. The gap delay and silent period were manipulated by changing the onset of stimulus presentation. The gap delay (between stimuli presentation and volume acquisition) was altered from 1 to 3.5 s by 0.1 s intervals. The TR was unaltered for the gap delay manipulations. The silent period durations were between ≈9 and 5 s. Periods of TR were for *TR* = 15 s to *TR* = 10 s.

In the second series of experiments, we used the optimized paradigm, but substituted the auditory stimuli with silent stimuli (Figure 1b right side panel). All aspects of the experimental design for optimization and for silent stimuli type I error rate assessment were identical. A false positive (type I error) was finding BOLD signal response for a voxel during the silent stimuli presentations. The final run paradigm for our sparse sampling of silence to ascertain type I errors consisted of the following parameters: *TR* = 15,000 ms, gap delay = 0.100 s, 3 s silent stimuli presentation, TA = 1.02 with 34 slice acquisition, Volume 1 (VL1 = 1.02 s), 1 s separation between volumes, Volume 2 (VL2 = 1.02 s), and the reaming time of the TR period, 8.86 s of silence. The silent paradigm was repeated for 74 blocks for two separate runs.

**FIGURE 2:**
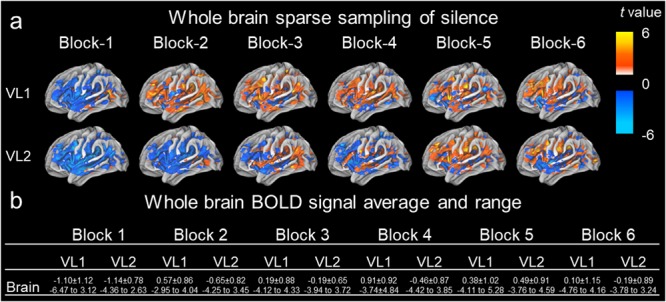
Whole brain sparse sampling of silence, derived BOLD signal average and range. A sparse sampling paradigm was conducted and whole brain activation/deactivation maps were derived for *t*-value difference from the auditory evoked paradigm (Figure 1a,b). **(a)** The entire brain activation/deactivation *t-*values were mapped (unthresholded) for each block (column) by the first volume (VL1) or second volume (VL2). The color bar denotes the *t*-value range for the paradigm. Here, the *t*-values were taken to derive type I errors. **(b)** The table represents average ± standard deviation and maximum to minimum *t-*values for the entire acquired volume.

**FIGURE 3:**
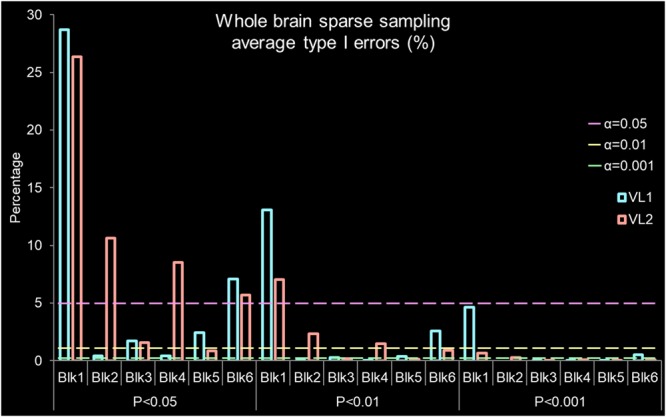
Whole brain sparse sampling of silence average type I error percentage derived from activation/deactivation maps (Figure 2a). The bars are observed type I errors from the blocks grouped by *P*-values. The x-axis is grouped volume, represented by VL1 (#98f5ff cadet blue) or VL2 (#ffa298 Salmon pink), by *P*-values (*P* < 0.05, *P* < 0.01, and *P* < 0.001) and by block (Blk). The y-axis is percentage of false positives found if accepting a specific alpha (α) value where the lines represent α = 0.05 (#ff98f5 light magenta), α = 0.01 (#f5ff98 light yellow), and α = 0.001 (#98ffa2 light green). The expected type I error rate α is found by dashed lines for α = 0.05, α = 0.01, and α = 0.001. Above these lines for an accepted *P*-value, a type I error has been committed. Therefore, rejecting the null hypothesis (indicating there is a difference) when no relevant BOLD activation/deactivation is present.

**FIGURE 4:**
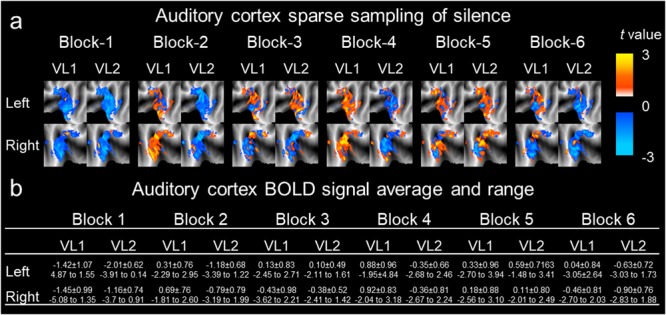
Auditory cortex sparse sampling of silence, derived BOLD signal average and range. A sparse sampling paradigm was conducted and auditory cortex activation/deactivation maps were derived for *t-*value difference during the auditory evoked paradigm (Figure 1a,b). **(a)** The entire auditory cortex activation/deactivation *t*-values were mapped (unthresholded) for each block (column), by the sub-column first volume (VL1) or second volume (VL2), and by the left and right hemisphere for each row of brains. The color bar denotes the *t*-value range for the paradigm. Here, the *t*-values were taken to derive type I errors. **(b)** The table represents average ± standard deviation and maximum to minimum *t*-values for the entire auditory cortex volume. Blocks are represented in columns with sub-column delineations for the first and second auditory cortex volume acquired. The left and right hemisphere is represented by the row on the table.

**FIGURE 5:**
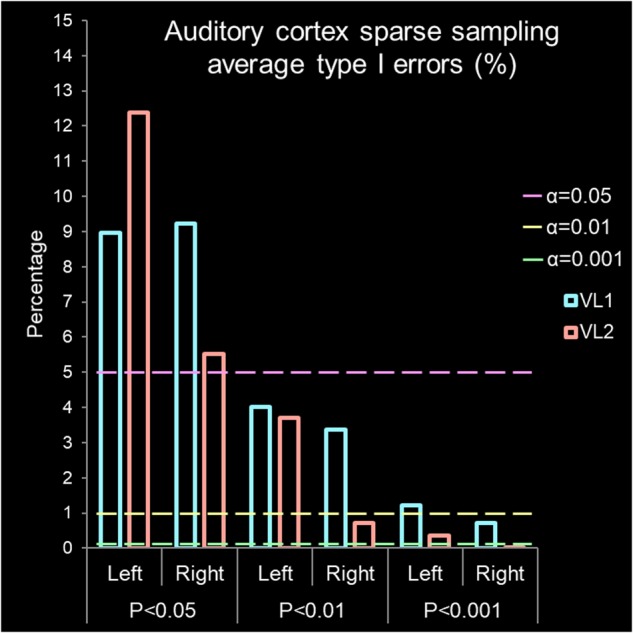
Auditory cortex sparse sampling of silence. Average type I error percentage derived from activation/deactivation maps (Figure 4a). The bars are observed type I errors from the left and right hemisphere grouped by *P-*values. The x-axis is grouped volume, represented by VL1 (#98f5ff cadet blue) or VL2 (#ffa298 Salmon pink) and by *P*-values (*P* < 0.05, *P* < 0.01, and *P* < 0.001). The y-axis is percentage of false positives found if accepting a specific alpha (α) value where the lines represent α = 0.05 (#ff98f5 light magenta), α = 0.01 (#f5ff98 light yellow), and α = 0.001 (#98ffa2 light green). The expected type I error α rate is found by dashed lines for α = 0.05, α = 0.01, and α = 0.001. Above these lines for an accepted *P*-value, a type I error has been committed. Therefore, rejecting the null hypothesis (indicating there is a difference) when no relevant BOLD activation/deactivation is present.

Our design matrix for analyzing the auditory sparse sampling data from the first series of experiments was to aggregate all alike stimuli events together (Norman-Haignere et al., 2013; see Figure 1b). Subsequently, silent stimuli were substituted, and an identical design matrix was implemented for the second series of experiments. For visualization in the figures, the average of 12 blocks with 2 blocks discarded (first and last) was presented in 6 blocks (74 blocks total). Volumes within a TR (Figure 1) were aggregated together by blocks (Blk) based on a generic auditory paradigm (Norman-Haignere et al., 2013; See Figure 1 for design; Figure 2–5; see Supplementary Tables SI1, SI2). Here we modeled the most robust HRF (Figure 1a) to 3 s auditory and silent stimuli (Figure 1b blue box). Normally, sound stimuli are modeled within 4–6 s after their presentation (Figure 1b) to capture the most robust BOLD signal (Edmister et al., 1999; Hall et al., 1999; Talavage et al., 1999; Gaab et al., 2003, 2007a,b, 2008; Bunzeck et al., 2005; Schwarzbauer et al., 2006; Zaehle et al., 2007; Schmidt et al., 2008; Liem et al., 2012). A two-volume cluster sparse sampling acquisition was employed with 1 s separation, *TR* = 15 s, and with different gap periods between the stimuli and volume acquisition (Figure 1c). Here we varied the model of the putative fMRI response for a BOLD signal (Figure 1a; red dashed line is the variation). A ROI pertaining to the auditory cortex was delineated (Figure 1d), based on a probabilistic map for Heschl’s gyrus (HG; Figure 1e; Morosan et al., 2001; Rademacher et al., 2001). Once the HG was extracted, we calculated average ROI activation and error rates (Figure 1f). We present the type I error rates associated with this activation.

### Image Processing

Image processing used FSL tools (fMRIB, University of Oxford, United Kingdom) using FEAT (FMRI Expert Analysis Tool) version 5.98. The general linear model (GLM) was used to assess the relationship between the sound or silent stimuli and the BOLD signal using the double gamma function convolved with the HRF (Leaver and Rauschecker, 2010). Functional volumes were preprocessed for motion correction, linear trend removal, spatial smoothing using a 5 mm FWHM Gaussian kernel, and elimination of low-frequency drifts using a temporal high-pass filter with a cutoff of 100 s. Preprocessing of the fMRI statistical maps included spatial realignment, coregistration with anatomical data using FSL FLIRT, spatial normalization and alignment with MNI 152 T1-weighted MRI scans. Further image analysis was performed using custom scripts in Matlab to segment ROI from the Jülich histological atlas (Morosan et al., 2001). For the first and second series of experiments, two different runs of 74 blocks were collected for a total run time of 18 min and 30 s. A 5 min rest period was given between the two runs. A fixed-effects analysis averaged the two runs and a mixed-effects higher level analysis was performed to average the data associated with the silent stimuli across subjects (*n* = 15). For the first series of experiments using auditory stimuli to optimize the paradigm, multiple comparisons for averaging volumes were controlled for by using random field theory. Here, a cluster-defining threshold (CDT) of *P* < 0.05 was used (voxel *z* > 2.3). For the second series of experiments using silence, finalized volumes were visualized and assessed by plotting all *t*-values by voxel data unthresholded, but using the same model as the auditory paradigm. The *t*-value by voxel for a certain α level was used to determine significance (unthresholded). Finalized volumes and ROI were mapped to the Conte69 atlas in MNI space using CARET v5.65 (Van Essen et al., 2012). Auditory stimuli, as described above, and actual silent stimuli devoid of fine structure and envelope sound information were delivered via Matlab (Statistics Toolbox Release 2012b, The MathWorks, Inc., Natick, MA, United States) with the Psychophysics Toolbox extension^1^ on a HP pc (Intel Core i5-4210U CPU at 2.40GHz) with a RealTek High Definition Audio card.

### Definitions

The *P*-value, assuming the null hypothesis is true, is the probability of obtaining a result as extreme or more extreme than the observation (Zar, 1999). The *P*-values for the present study were *P* < 0.05, *P* < 0.01, *P* < 0.001. Type I error (α), assuming the null hypothesis is false, is the probability of making this error. That is, rejecting the null hypothesis when it is true (Zar, 1999). The type I error (α) for the present study were α = 0.05, α = 0.01, α = 0.001. Therefore, inference for significance for the present manuscript was performed using voxels passing specific α levels (α = 0.05, α = 0.01, α = 0.001). Above these levels for a specific *P*-value was erroneously concluding BOLD activation/deactivation was present, when no activation/deactivation (i.e., modulation) should occur. Were presented results in percentages associated with the false positive conclusion.

## Results

Figure 1 presents a summary of the experimental design. Table 1 presents summary findings of *P*-value by type I error percentage. Figure 2 presents unthresholded *t*-values for the whole brain sparse sampling of silence analysis. Figure 3 presents the whole brain sparse sampling of silence average type I error percentage by *P*-value for a specific α level. Figure 4 presents unthresholded *t*-values for the auditory cortex sparse sampling of silence ROI analysis. Figure 5 presents the auditory cortex sparse sampling of silence average type I error by *P*-value for a specific α level. Supplementary Table SI1 presents the whole brain sparse sampling of silence average type I error percentage by *P*-value for each Block by VL1 and VL2. Supplementary Table SI2 presents the auditory cortex sparse sampling of silence average type I error percentage by *P*-value for each Block by VL1 and VL2 for left and right hemisphere.

**Table 1:** Summary findings of *P*-value by type I error percentage for whole brain and auditory cortex.

*P*-value threshold		*P* < 0.05	*P* < 0.01	*P* < 0.001
Whole brain	VL1	6.813% ± 10.047%	2.735% ± 4.714%	0.873% ± 1.691%
	VL2	8.939% ± 8.529%	2.005% ± 2.369%	0.1878% ± 0.226%
Auditory cortex	VL1	9.088% ± 0.137%	3.692% ± 0.327%	0.971% ± 0.254%
	VL2	8.948% ± 3.440%	2.201% ± 1.494%	0.185% ± 0.162%

*Errors for *P* < 0.05, *P* < 0.01, and *P* < 0.001, are higher than acceptable by a factor of 1.69, 2.66, and 5.54, respectively, than the α level. The difference between whole brain and auditory cortex errors for *P* < 0.05, *P* < 0.01, and *P* < 0.001, were 1.14, 0.58, and 0.05%, respectively greater for the auditory cortex.*

### General BOLD Signal Activation/Deactivation

The average BOLD signal was calculated separately for the left and right hemisphere modeled on silent stimuli (Figure 1b). Left and right hemispheres of the primary auditory cortex where delineated separately (Figure 1d). No difference was found between the first or second volume acquisitions (Table 1). The left and right hemisphere *t*-value was non-significantly different when assessing the run by block (*P* = 0.587, *t* = 0.569 and *P* = 0.376, *t* = 0.945, respectively). Although considerable activation for some time-points can be visualized (i.e., moving across a block Figure 2a), the average activation was non-significant. The difference between the left and right hemisphere assessing the run by block was non-significant (*P* = 0.358, *t* = 0.985, *df* = 1.7; Figure 2a); nevertheless, the right hemisphere had greater activation over the left. The mean absolute *t*-value difference in BOLD activation between the left and right hemisphere was *t* = -0.128, with 95% confidence interval of this difference: from *t* = -0.435 to 0.179, respectively. Average left and right hemisphere activation over the run was *t* = 0.143 ± 0.709 and *t* = 0.271 ± 0.810. Here we presented the left hemisphere view for ease of visualization (Figure 2a).

### Whole Brain Type I Errors

Figure 2a demonstrates the activation/deactivation maps by block and volume for the entire brain. Figure 2b is a table of the average and range of BOLD signal by block and volume. The average whole brain BOLD signal *t*-value for the all the blocks was -0.168 ± 0.929 (SD), 0.003 (sem). The whole brain range of activation/deactivation was minimum *t*-value = -4.398 ± 0.803 SD to maximum *t*-value 3.879 ± 0.731 SD. A paired *t*-test of the average change in activation/deactivation by volume acquisition within a block to determine if the first or second volume was different, was non-significant (*t*_5_ = 2.129, *P* = 0.087, mean difference 0.541, CI: -0.113 to 1.195, correlation coefficient *r* = 0.514, *P* = 0.148). Therefore, the first volume acquisition was not significantly different from the second volume acquisition (Table 1). Nevertheless, note the wide range in activation and deactivation values (Figure 2b). The average number of voxels analyzed was 105,543 ± 1,907. Figure 3 demonstrates the average type I errors. The following are the average combined left/right hemisphere percent false positives. The average number of false positives for *P* < 0.05 were for VL1, 6.813% ± 10.047%, and for VL2, 8.939% ± 8.529%. The average number of false positives for *P* < 0.01 were for VL1 = 2.735% ± 4.714%, and for VL2 = 2.005% ± 2.369%. Accepting a more conservative probability *P* < 0.001, the average number of false positives for VL1 = 0.873% ± 1.691% and for VL2 = 0.188% ± 0.226%.

### Auditory Cortex Type I Errors

Figure 4a demonstrates the activation/deactivation maps by block and volume for the left and right auditory cortex. Figure 4b is a table of the average and range of BOLD signal by block and volume for the left and right auditory cortex. For VL1 left hemisphere auditory cortex, the average BOLD signal *t*-value for all the blocks was 0.047 ± 0.908 (SD), 0.017 (sem). For VL1 left hemisphere auditory cortex, the minimum and maximum *t*-values were -2.891 and 3.109. For VL1 right hemisphere auditory cortex, the average BOLD signal *t*-value for all the blocks was -0.090 ± 0.870 (SD), 0.019 (sem). For VL1 right hemisphere auditory cortex, minimum and maximum *t*-values were -2.9717 and 2.416. For VL2 left hemisphere auditory cortex, the average BOLD signal *t*-value for the all the blocks was -0.583 ± 0.651 (SD), 0.012 (sem). For VL2 left hemisphere auditory cortex, minimum and maximum *t*-values were -2.771 and 1.767. For VL2 right hemisphere auditory cortex, the average BOLD signal *t*-value for all the blocks was -0.584 ± 0.741 (SD), 0.016 (sem). For VL2 right hemisphere auditory cortex, minimum and maximum *t*-values were -2.820 and 1.826. A paired *t*-test of the average change in activation/deactivation by volume acquisition within a block, to determine if the first or second volume was different, was significant (*t*_12_ = 2.863, *p* = 0.015, mean difference -0.562, CI: -0.994 to -0.130, correlation coefficient *r* = 0.593, *P* = 0.021). Therefore, the first volume acquisition was significantly different from the second volume acquisition. Nevertheless, accepting a more conservative probability *P* < 0.01, the volumes were not different. Note the wide range in activation and deactivation values (Figure 4b).

The first volume from the left and right auditory cortex were not significantly different (*t*_6_ = 0.950, *P* = 0.386, mean difference 0.138 CI: -0.235 to 0.511, correlation coefficient *r* = 0.915, *P* = 0.005). For VL1, left and right hemisphere were highly significantly correlated in their BOLD signal response. For the second volume, the left and right auditory cortex were not significantly different (*t*_6_ = 0.002, *P* = 0.998, mean difference 0.001 CI: -0.560 to 0.561, correlation coefficient *r* = 0.926, *P* = 0.004). For VL2, left and right hemisphere were highly significantly correlated in their BOLD signal response. Note the wide range in activation and deactivation values (Figure 4b). The average number of voxels analyzed was 2,767 and 2,040 for the left and right hemisphere, respectively. Figure 5 demonstrates the average type I errors. The following are the average combined left/right hemisphere percent false positives. The average number of false positives for *P* < 0.05 was for VL1 = 9.088% ± 0.137%, and for VL2 = 8.948% ± 3.44%. The average number of false positives for *P* < 0.01 was for VL1 = 3.692% ± 0.327% and for VL2 = 2.201% ± 1.494%. Accepting a more conservative probability *P* < 0.001, the average number of false positives was for VL1 = 0.971% ± 0.254% and for VL2 = 0.185% ± 0.162%.

## Discussion

The present study analyzed type I errors during a ssfMRI paradigm using silent stimuli with the null hypothesis of no BOLD response. Here, we sought to determine if auditory cortex activation/deactivation could be modeled by a HRF in a sparse sampling paradigm using silent stimuli, where no auditory task existed. We report type I errors associated with sparse sampling of silence in the whole brain and in the ROI most commonly used during ssfMRI, the primary auditory cortex (Table 1). These errors result in false positives, rejecting the null hypothesis in favor of the alternative hypothesis, when this conclusion is false. Similar error rates are distributed evenly across the brain and primary auditory cortex. The present study recommends conducting further assessments in ssfMRI paradigms such as FDRs and effect size statistics. Conservative statistical inference for fMRI should be used in order not to breach the assumptions of the underlying the tests.

### Acoustic Noise During fMRI

Acoustic scanner noise results from the gradient magnetic field and radiofrequency pulses used to generate sequences for scanning (Hoiting, 2005). The most common pulse sequences used in fMRI, such as echo planar imaging (EPI), consist of the fast succession of alternating readout and phase encoding gradient currents which result in high amplitude and frequency acoustic noise (Moelker and Pattynama, 2003). The current study optimized the ssfMRI to minimize acoustic scanner noise contributions to the BOLD signal during the auditory paradigm. After the optimization, silence was substituted for auditory stimuli and here the experiment examined the null hypothesis of no effect (Figure 1b right side). No difference in BOLD signal was found by hemisphere assessing the run by block of volume acquisitions. No difference in BOLD signal was found between volume acquisitions (VL1 and VL2). Despite these findings, high false positives were found (Table 1), which are unlikely due to acoustic noise, but rather aspects of the resting state BOLD response (i.e., undershoot or overshoot), processing steps, or breaching the assumptions of the model. Normally, the gradient currents are the primary sources of acoustic noise during MRI (McJury and Shellock, 2000; Moelker and Pattynama, 2003; Hoiting, 2005). It is the task of a ssfMRI acquisition to minimize acoustic scanner noise contributions to the auditory paradigm. Different acquisition paradigms are employed to optimize the BOLD response to the auditory task under investigation (Amaro et al., 2002). Most protocols intersperse periods of silence in a block design (Amaro et al., 2002; Liem et al., 2012) to maximize the volume acquisition to when the BOLD signal is strongest (Perrachione and Ghosh, 2013). In the present protocol, we were interested in type I errors associated with the null hypothesis of no BOLD activation. While we cannot completely rule out background scanner noise contributions, the present study took care to eliminate these artifacts by devising a protocol with a long TR (here TR = 15 s) to allow the BOLD signal to reach baseline before acquiring a subsequent volume.

### Scanner Noise Inducing Auditory Activation

Ulmer et al. (1998) mimicked the MRI environment by using taped scanner noise consisting of 60–80 db (decibels, peak tone frequencies ranging between 500 and 4,000 Hz) delivered in four 20 s intervals alternating with five 20 s “rest” intervals. The study by Ulmer et al. (1998) found significant activation within the right or left transverse temporal gyrus, planum polare, planum tempolare, middle temporal gyrus and superior temporal sulcus. Bandettini et al. (1998) found the hemodynamic response signal falls from about 7% to zero in the first 5–7 s after acoustic noise (i.e., gradients). With a *TR* = 9 s using 100 ms syllables and 4-slice, 16-slice, and 64-slice acquisitions, Shah et al. (1999) found a greater number of activated voxels in the auditory cortex during the quieter periods of the 4-slice and 16-slice. This was likely because more target syllables fell in the quiet periods. Di Salle et al. (2001) determined that the BOLD signal reached a stable baseline within the primary auditory cortex approximately 4–5 s after stimulation with 10 s sine tones at 1,000 Hz, amplitude-modulated 10 Hz square waves (rise/fall time = 5 ms, plateau = 40 ms, duty cycle = 0.5, interstimulus interval = 50 ms, output 70 sb SPL). Interestingly, they noted in one pattern of response, a BOLD signal decay continued with a prolonged undershoot until the end of the sampling period (Di Salle et al., 2001). Zaehle et al. (2007) and Schmidt et al. (2008) compared continuous acquisition with clustered acquisition and found significantly greater activation in primary auditory regions (Heschl’s gyrus, planum polare and planum temporale of each hemisphere) during the clustered temporal acquisition compared to the sparse (single volume) paradigm. The purposes of sparse sampling is to minimize acoustic noise contributions to the BOLD signal; nevertheless, we note the model still results in high false positives at the traditional *P* < 0.05 level. These errors could be due to aspects of the BOLD signal, underlying assumptions of the model being breached, or aspects in processing images which introduce noise. We note, realigning individual subject data to correct for motion during scanning, transforming images, resampling estimates of the signal, smoothing which involves averaging voxels (Eklund et al., 2016; Cox et al., 2017a,b; Flandin and Friston, 2017) and increases spatial correlation, registration to a common template (normalization; Mueller et al., 2017) etc., all preprocessing steps could potentially cause errors and introduce noise (see textbooks for review: Frackowiak et al., 2003; Buxton, 2009).

### Optimizing BOLD Signal in ssfMRI Paradigms

The first sparse sampling protocols where Hall et al. (1999) acquiring a volume every *TR* = 14 s or Edmister et al. (1999) acquiring a volume every *TR* = 8 s. Perrachione and Ghosh (2013) recommended a “sweet spot” *TR* = 6 s per volume. Further, Bunzeck et al. (2005) and Schwarzbauer et al. (2006) acquired a series of 5 volumes per TR, and Zaehle et al. (2007), Schmidt et al. (2008), and Liem et al. (2012) acquired a series of 3 volumes per TR. This process was termed clustered sampling (Gaab et al., 2003, 2007a,b, 2008). These protocols revealed the underlying contributions to scanner noise when modeling the noise of the scanner as the underlying explanatory variable. The present study modeled a non-existent 3 s silent stimuli as the explanatory variable and we found significant BOLD signal present. The high false positives we reported could be due to our model assessing different aspects of the BOLD response (based on the delay ∼4–6 s; Perrachione and Ghosh, 2013). For example, using the finger tapping task, the BOLD signal time course at the cortical surface had a stronger overshoot after the task onset and a stronger undershoot proceeding the task offset (Huber et al., 2015). We cannot rule out our optimized paradigm was assessing aspects of the overshoot or undershoot features of BOLD. Here, an assumed false-positive would be confounded (i.e., overestimated) due to the remaining BOLD signal. These factors such as designing adequate TR to capture the BOLD response at its height, could mean aspects of the overshoot or the prolonged post-stimulus undershoot are contributing to type I errors in the current paradigm. To design a better paradigm, the overshoot and prolonged post-stimulus undershoot should be considered to estimate these features of a stimulus response (van Zijl et al., 2012).

### Type I Errors in fMRI Paradigms

Several studies utilizing fMRI have highlighted the errors associated with using non-conservative α-values or rejecting the null hypothesis in favor of the alternative, due to paradigm design (Bennett et al., 2010; Fisher and Student, 2012). Broadly, a previous critique has recommended reporting effect size estimations of the measure of interest, conducting sample size statistics to protect against trivial effects, and using conservative hypothesis testing (Friston, 2012, 2013; Ingre, 2013; Lindquist et al., 2013). The present study chose a common sample size (*n* = 15) and different *P*-values to explore type I errors in ssfMRI data. Here, we found that to minimize type I errors and ensure sufficient power of the study, a more conservative α level needs to be utilized (Benjamin et al., 2018; Trafimov et al., 2018). Nevertheless, there is an ongoing debate as to whether it is a valid assumption to changing the alpha level (as an example, α = 0.05 to α = 0.01) used for determining significance to reduce false positives (Benjamin et al., 2018; Trafimov et al., 2018). Controlling for false positive (Genovese et al., 2002), and using a threshold to specify a large cluster of voxels (Friston et al., 1996), can be methods to ensure conservative data reporting. The most in-depth study to-date found family-wise error rates (FWER) for cluster-wise inference far exceeded their nominal 5% level, whereas voxel-wise inferences were valid, but conservative, often falling below 5% (Eklund et al., 2016). Reassessments of the Eklund et al. (2016), for example, using the non-parametric FDR-based method, found that using CDT = 0.001 and RFT–FWE correction was “trustworthy” whereas a CDT = 0.01 depended on the corrected *P*-value (Kessler et al., 2017). In the present study, we analyzed voxel-wise error rates and found that in ssfMRI, they were far higher than expected by approximately 4% for *P* < 0.05. Future studies need to conduct preliminary assessments using robust experimental designs with clear hypothesis statements. Alternative methods such the FDR can be implemented, which assumes false positives will be detected and controls that type I errors make up no more than α of the discoveries (Genovese et al., 2002; Schwartzman et al., 2009). The FDR is defined as the proportion of false positives among all rejected tests (Lindquist and Mejia, 2015), therefore is done after deriving the imaging data, i.e., *post hoc*. Changing α level and using more conservative *P*-values are steps implemented prior to deriving your functional maps. Each of these approaches could be implemented, and a recent review recommends using both FWER and FDR (Lindquist and Mejia, 2015).

### Study Limitations and Future Directions

The limitations of the protocol were methodological. There were two volume acquisitions separated in time by 1 s. Our model was based on a 3 s stimuli contained within a TR = 15 s. The second acquired volume due to the RF pulse of the first volume was not acquired in a fully relaxed state, which could potentially contribute to second volume BOLD signal and errors. We note, activation and type I errors were not significantly different between volumes for the whole brain or auditory cortex. Nevertheless, during different sparse sampling paradigms this could be an issue. The optimized model employed could be assessing different aspects of the undershoot or overshoot (Perrachione and Ghosh, 2013). For example, there could be a chance for all the BOLD signal to be outside the model, if during the optimization this occurred for all the averaged blocks of the entire fMRI run. Here the model could miss the peak HRF and be modeling undershoot or overshoot. This would overestimate or underestimate the BOLD signal to silence causing false positives. Although this is unlikely, if the flawed optimization to the HRF were consistent across the 18 min and 30 s run, it could confound the results significantly. Future studies should examine different silent stimuli lengths with different TRs and assess scanning sequences such as continuous or interleaved paradigms with a greater number of volume acquisitions. For example, Mueller et al. (2011) used 10 s musical stimuli to determine the difference between continuous (36 slice, *TR* = 2.5 s, TE = 30 ms, *TA* = 1.08 s), sparse sampling (15 slice, *TR* = 11 s, *TE* = 29 ms, *TA* = 0.435 s) and interleaved silent steady fMRI (5 sequential volumes, 15 slice, *TR* = 15 s, *TE* = 27 ms, *TA* = 0.405 s). The authors indicated interleaved silent steady fMRI provided increased sensitivity compared to continuous and sparse sampling for auditory stimuli (Mueller et al., 2011). We note, no difference was found between the first and second volume in the present paradigm (Figure 2, 4). The paradigm was not considered a pure sparse sampling (one volume acquisition), nor a pure interleaved silent steady state paradigm (several volume acquisitions), since 2 volumes were acquired. Mueller et al. (2011) had 5 volumes per stimuli and a long duration auditory stimulus was used (10 s). Here we used a short stimulus (3 s) and our two volume acquisitions based on ∼4–6 s delay of the BOLD signal (Perrachione and Ghosh, 2013), and our optimization procedure. With a greater number of volume acquisitions, we would anticipate only a slight difference between volume responses (Figure 2, 4), therefore, similar type I error rates. Lastly, because we delivered actual silent stimuli via the Psychophysics Toolbox in Matlab. Here, the on/off pressure wave of queuing the auditory signal, when no-auditory signal existed, could have elicited the activation witnessed due to the ramp up function in the MRI-compatible headphones (AudioSystem, Nordic NeuroLab). However, due to the noise attenuation headphones (30 db) being equivalent to background noise in the MRI environment (≈29 db), it is unlikely (see Hoiting, 2005 for review of acoustic noise contributions in the MRI environment).

## Data Availability

Data is uploaded to www.fmanno.com and https://www.nitrc.org/ projects/sparse_2018.

## Ethics Statement

The research protocol was approved by the Ethics Committee on the Use of Humans as Experimental Subjects in accordance with the Declaration of Helsinki, 2013.

## Author Contributions

FM, JF-R, and FB designed the research. FM and FB performed the research. FM analyzed the data. FM, JF-R, SM, SC, CL, and FB wrote the manuscript.

## Conflict of Interest Statement

The authors declare that the research was conducted in the absence of any commercial or financial relationships that could be construed as a potential conflict of interest.

## Footnotes

1 http://psychtoolbox.org/

## Supplementary Material

TABLE SI1 Type I errors observed for *P*-values for whole brain. Blocks are organized by column with sub-column delineations for the first volume (VL1) or second volume (VL2) and rows for *P*-value. The number of voxels within the entire brain exhibiting type I error are indicated by 30,894 of 107,598 voxels, for example as found in Block 1, VL1 for *P* < 0.05. Below the number of voxels is the percentage of type I errors found for the specific *P*-value.

TABLE SI2 Type I errors observed for *P*-values within auditory cortex. Blocks are organized by column with sub-column delineations for volumes (VL1 and VL2) and rows for left and right hemisphere auditory cortex parcellation. The number of voxels within auditory cortex exhibiting type I error are indicated by, for example, 839 of 2767 voxels found in Block 1, VL1 left hemisphere for *P* < 0.05. Below the number of voxels is the percentage of type I errors found for the specific *P*-value.
