# Supplementary figures and images for: Sparse Sampling of Silence Type I Errors With an Emphasis on Primary Auditory Cortex
Source: Front Neurosci. 2019 May 31;13:516. doi: 10.3389/fnins.2019.00516 (PMC6554478; doi:10.3389/fnins.2019.00516)

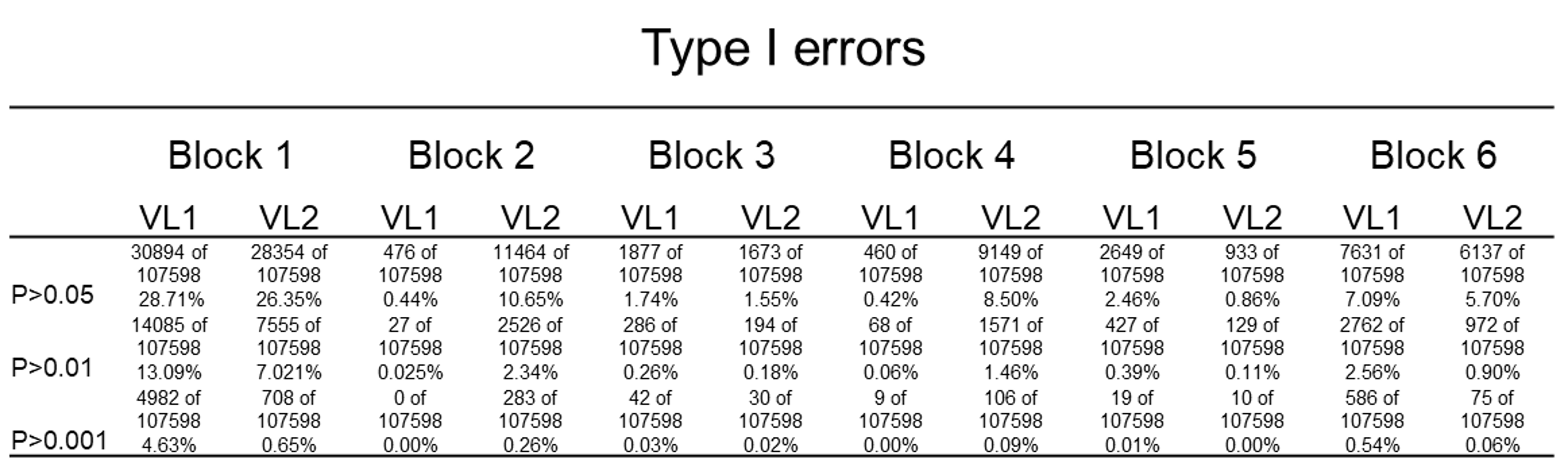

Supplement: TABLE SI1 — Type I errors observed for P-values for whole brain. Blocks are organized by column with sub-column delineations for the first volume (VL1) or second volume (VL2) and rows for P-value. The number of voxels within the entire brain exhibiting type I error are indicated by 30,894 of 107,598 voxels, for example as found in Block 1, VL1 for P < 0.05. Below the number of voxels is the percentage of type I errors found for the specific P-value. [file Image_1.tiff]

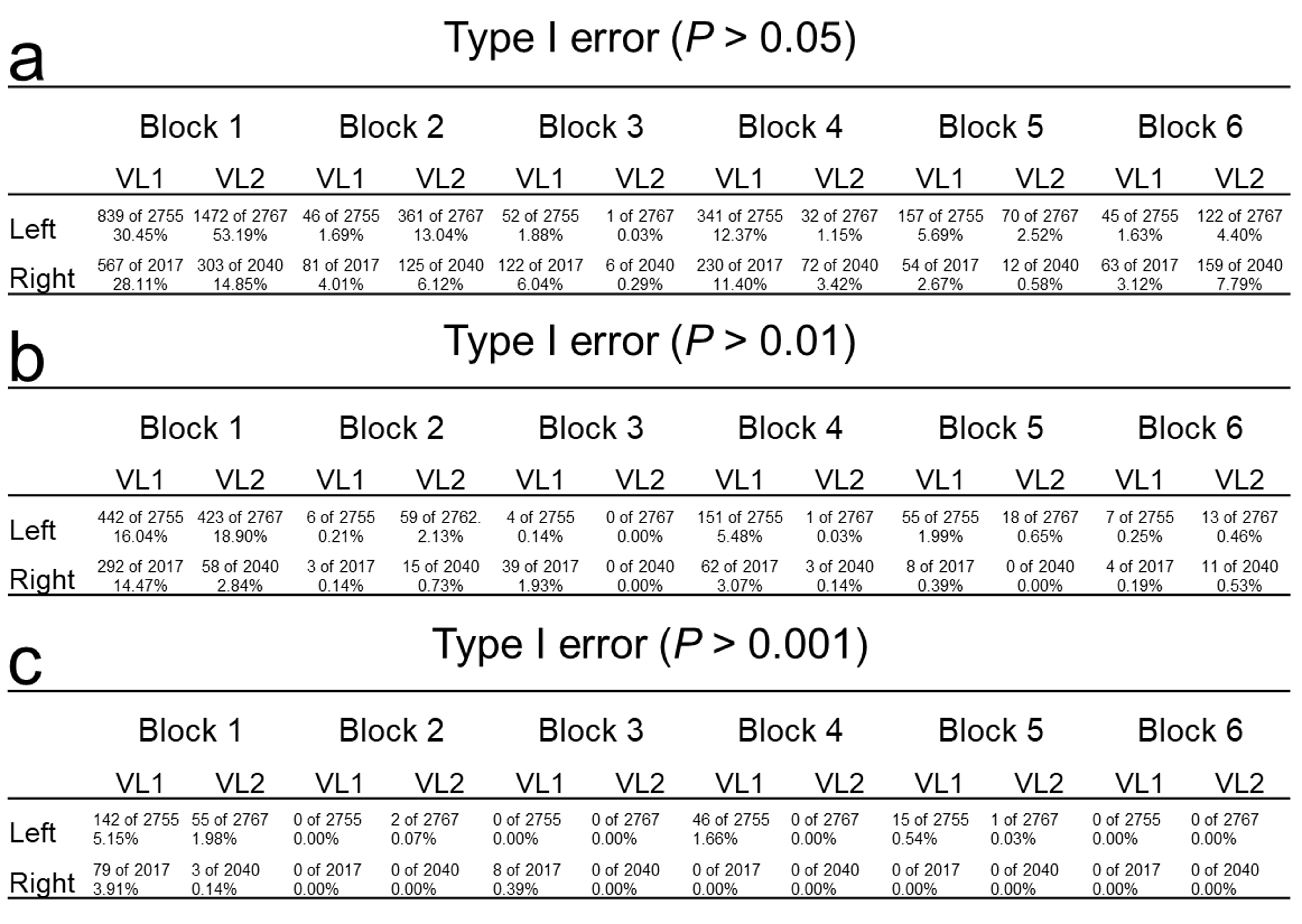

Supplement: TABLE SI2 — Type I errors observed for P-values within auditory cortex. Blocks are organized by column with sub-column delineations for volumes (VL1 and VL2) and rows for left and right hemisphere auditory cortex parcellation. The number of voxels within auditory cortex exhibiting type I error are indicated by, for example, 839 of 2767 voxels found in Block 1, VL1 left hemisphere for P < 0.05. Below the number of voxels is the percentage of type I errors found for the specific P-value. [file Image_2.tiff]
